# Understanding the science of portion control and the art of downsizing

**DOI:** 10.1017/S0029665118000435

**Published:** 2018-05-24

**Authors:** Marion M. Hetherington, Pam Blundell-Birtill, Samantha J. Caton, Joanne E. Cecil, Charlotte E. Evans, Barbara J. Rolls, Tang Tang

**Affiliations:** 1 School of Psychology, University of Leeds, Leeds LS2 9JT, UK; 2 School of Health and Related Research, Section of Public Health, University of Sheffield, Sheffield S1 4DA, UK; 3 School of Medicine, University of St Andrews, St Andrews KY16 9TF, UK; 4 School of Food Science and Nutrition, University of Leeds, Leeds LS2 9JT, UK; 5 Nutritional Sciences, The Pennsylvania State University, Pennsylvania PA 16802, USA; 6 School of Design, University of Leeds, Leeds LS2 9JT, UK

**Keywords:** Portion size, Food intake, Children, Adolescents, Energy density

## Abstract

Offering large portions of high-energy-dense (HED) foods increases overall intake in children and adults. This is known as the portion size effect (PSE). It is robust, reliable and enduring. Over time, the PSE may facilitate overeating and ultimately positive energy balance. Therefore, it is important to understand what drives the PSE and what might be done to counter the effects of an environment promoting large portions, especially in children. Explanations for the PSE are many and diverse, ranging from consumer error in estimating portion size to simple heuristics such as cleaning the plate or eating in accordance with consumption norms. However, individual characteristics and hedonic processes influence the PSE, suggesting a more complex explanation than error or heuristics. Here PSE studies are reviewed to identify interventions that can be used to downsize portions of HED foods, with a focus on children who are still learning about social norms for portion size. Although the scientific evidence for the PSE is robust, there is still a need for creative downsizing solutions to facilitate portion control as children and adolescents establish their eating habits.

The purpose of this review is to identify potential drivers of the portion size effect (PSE), where large portions of foods and beverages encourage large intakes, and to identify possible strategies to moderate the PSE. In particular, strategies to promote downsizing in children are considered, since there is more scope for children to learn about portion control and to establish portion norms during development.

It has been shown that portion sizes of products offered in the marketplace have increased over time in the USA, Europe and Australia^(^^1^^–^^3^^)^. At the same time as portion sizes served inside and outside the home have increased, population-based studies have shown an increase in levels of overweight and obesity^(^^1^^)^. In 2014, the WHO suggested that limiting portion sizes to reduce overall energy intake could reduce the risk of unhealthy weight gain^(^^4^^)^. The WHO has linked consumption of large portion sizes with overweight and obesity, but this does not infer causality. Most empirical evidence relating portion size to overconsumption has been gathered from laboratory-based experiments where portion sizes of foods and beverages can be manipulated systematically and the effects on intake measured^(^^5^^)^. In studies conducted by Rolls *et al*., providing large portions of high-energy density (HED) foods promoted greater energy intake relative to smaller portions^(^^6^^–^^11^^)^. These studies show that the PSE is a reliable, robust and enduring phenomenon observed across meal and snack items, age groups and contexts^(^^12^^)^.

It has been estimated using meta-analysis of sixty-five studies involving 109 separate observations that doubling the amount of food offered in laboratory-based studies results in an increase of approximately 35 % in amount eaten, and the relationship is curvilinear^(^^13^^)^. This indicates that there are limits to the PSE; it is not a simple dose–response relationship with a simple linear increase matched to large portions. Instead, at the upper limits of portion sizes, food intake is constrained by gastric fullness, internal signals of satiation and what is deemed an appropriate amount to eat, namely consumption norms^(^^13^^)^. According to this systematic review, the PSE appears to be weaker in children, women and individuals with overweight^(^^13^^)^. However, this conclusion is contested since careful laboratory investigations show that children with overweight are more responsive to large portions^(^^14^^)^. In adults, the results are mixed for the association between the PSE and BMI. In young adults, there is a relationship between high BMI and large portion sizes for HED foods^(^^15^^)^, but in other studies, the PSE is similar across weight categories^(^^12^^,^^16^^)^. These mixed results in adults may depend upon the types of foods offered. For example, when foods are ranked highly for taste, the PSE is amplified^(^^12^^)^.

To examine social and personal norms for portion sizes and the role of liking in these, Lewis *et al.*^(^^17^^)^ developed a computer-based task displaying twelve commonly consumed foods (e.g. fruit, cereal, pasta, biscuits, chocolate) presented in seventeen different portion sizes. Here the smallest size was a quarter of the standard UK reference portion size and the largest, four times the standard. Overall, portion size norms for all foods were greater than the standard UK reference. In particular, personal norms exceeded social norms for foods that were highly liked. Among males, unrestrained eaters and persons with obesity, personal norms were larger than social norms. While consumers appeared to recognise social norms for these commonly eaten foods, this was influenced by how much the food was liked^(^^17^^)^.

Many foods that are highly liked are HED including sweet snacks such as biscuits and cakes, or salty snacks including potato crisps. These are the types of foods that have been shown to be overconsumed when offered in large portions in a laboratory setting^(^^18^^)^. In general, HED foods tend to be more palatable than foods low in energy density (LED). If HED is defined as foods and beverages containing >10·4 kJ/g (2·5 kcal/g) and LED as items containing <10·4 kJ/g (2·5 kcal/g)^(^^19^^)^, then it would be expected that intake of foods and beverages higher in energy density will be associated with total energy intake and BMI. In the study of British adolescents, Albar *et al.*^(^^19^^)^ demonstrated a positive association between total energy intake and BMI, and portion sizes of a number of HED foods (biscuits, cheese, cream and cakes) were found to be positively associated with BMI in adolescents (adjusting for underreporting). They estimated that for each additional 418·4 kJ (100 kcal) increase in total energy intake, BMI increased by 0·19 kg/m^2(^^19^^)^, and suggested that adolescents could benefit from greater awareness of portion sizes of energy-dense foods to promote healthy eating and weight. However, it should be noted that this was based on cross-sectional data that do not infer causality.

Short-term laboratory studies of the PSE reveal that it is robust and reliable (represented schematically in Fig. 1)^(^^6^^–^^13^^,^^16^^,^^18^^,^^20^^)^. When large portions are offered, excessive amounts are eaten, and when foods are presented in large amounts, personal norms for portion size exceed social norms particularly if the foods are highly liked. The PSE is influenced by energy density and palatability^(^^12^^)^. To establish a link between portion size and positive energy balance, studies have been conducted beyond single meal occasions and over sufficient time to track body weight change following manipulations of portion size.

**Fig. 1.**
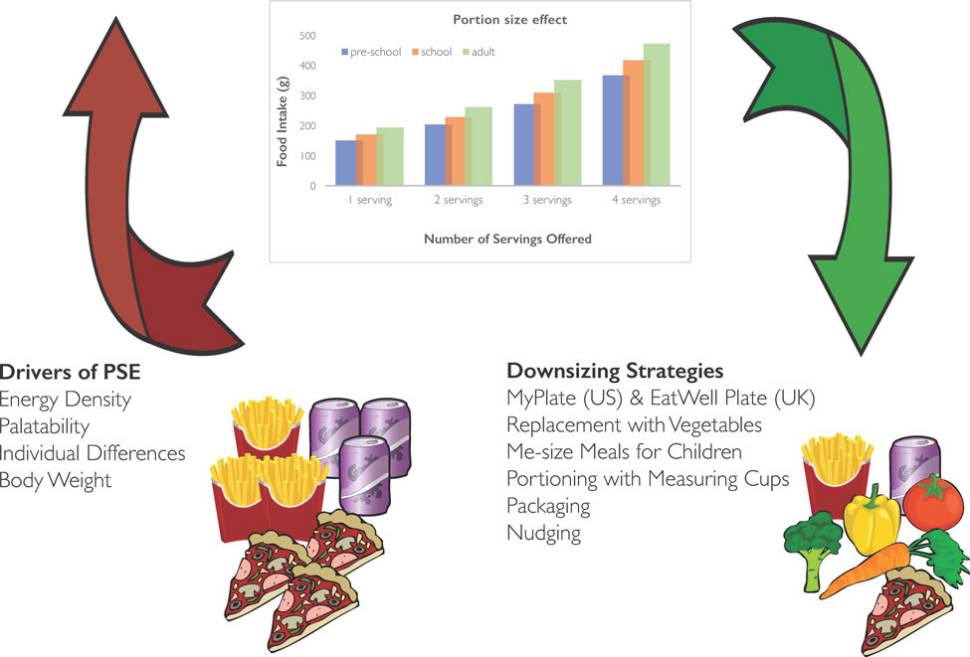
(Colour online) A schematic representation to illustrate a notional portion size effect (PSE) imagined from combining a number of laboratory studies (more offered, more eaten across age groups) as well as potential drivers (red) of the PSE and potential downsizing strategies (green) which could be applied to resist the PSE.

## Long-term effects of the portion size effect

Rolls *et al*. have shown that large portions presented consistently over 2 or 11 d increased food intake, producing an additional energy intake of 1769·8 kJ (423 kcal) daily^(^^18^^,^^20^^)^. Theoretically, if 1 kg body fat represents 32·2 kJ (7·7 kcal) stored energy, then additional energy intake of about 1769·8 kJ/d (423 kcal/d) would produce an increase in body weight of at least 1 kg in 18 d if adjustment were not made elsewhere in the diet or by increasing physical activity. If portion size is relevant to energy balance, then systematically increasing portion sizes could result in weight gain over time if there is no compensatory response to the excess energy intake. There are few controlled studies of long-enough duration to determine the effects of the PSE on body weight. In part, this may be due to the challenge of following participants over a period of time sufficiently long to assess changes in weight, at the same time offering large portions of food in a systematic way. One such study was conducted by French *et al.*^(^^21^^)^ within a work place setting. The investigators followed employees over a 6-month period randomising 233 of them to receive a boxed lunch each day consisting of a small 1674·6 kJ (400 kcal), intermediate 3347·2 kJ (800 kcal) or large 6694·4 kJ (1600 kcal) lunch or no boxed lunch in the control group. Energy intake at lunch and total energy intake across the day were significantly higher in the large lunch condition compared with the other conditions. However, weight gain was not different across the experimental groups. In the large lunch condition, the weight gain of about 1 kg was similar to that of the control group; whereas in the smaller boxed lunch conditions, weight was stable^(^^21^^)^. One interpretation of this outcome is that smaller lunches helped participants to maintain body weight. Participants in the control group and large lunch condition would need to compensate elsewhere in their diet or make adjustments to their physical activity levels to counter the effects of the PSE (large lunch condition) or the obesogenic environment (control group). Alternatively, the average weight gains across groups may mask individual differences in susceptibility to weight change, including response to large portions. The evidence that large portions promote overconsumption is convincing but the evidence that large portions directly promote weight gain is less clear. A creative, if obvious, solution to counter the PSE is to offer and to normalise smaller portion sizes at lunch since this appears to have helped consumers in the workplace investigation to defend against weight gain.

Overall, systematically offering large portions over time promotes increased energy intake, but it is more difficult to demonstrate specific, causal relationships between providing large portion sizes over time and weight gain in controlled studies. This could be attributed, in part, to individual differences in susceptibility to the PSE.

## Individual differences in portion size effect

In studies where individual characteristics have been measured, some participants responded to the presence of palatable foods in large portions to a greater extent than others^(^^13^^–^^17^^,^^19^^)^. In adults, few characteristics have clearly defined who is most susceptible to the PSE. In contrast, children's eating traits such as satiety responsiveness, food responsiveness and enjoyment of food have been related to the PSE. These eating traits are measured through parental reports on validated instruments such as the child eating behaviour questionnaire^(^^22^^)^. They are highly heritable^(^^23^^)^ and influence food intake including response to portion size. For instance, it has been demonstrated that children described as low in satiety responsiveness but high in food responsiveness were more sensitive to portion manipulations^(^^15^^)^. Interestingly, when both portion size and energy density were manipulated, it was found that children with high food responsiveness demonstrated a greater PSE than those with low scores on this eating trait^(^^24^^)^. In this same study, children with high enjoyment of food ate more of the LED food items than the HED items, indicating that this trait extends to all foods and not simply those HED or high in palatability.

These eating tendencies can be studied more systematically through objective measures including brain responses to food cues. Brain imaging during exposure to the images of food permits a more detailed investigation of automatic, unconscious responses to HED and LED foods^(^^25^^)^. For example, children with eating traits such as high enjoyment of food and food responsiveness when presented with food cues reveal differential brain activation when portions are served in large or small amounts. This has been tested in children aged 7–11 years and results showed that there are distinct areas of the brain responsive to these food cues^(^^25^^)^. Activation in areas of the brain thought to be involved in inhibitory control and information processing were reduced when large portions were viewed compared with small portions. This may provide an explanation, in part, for poor cognitive control around large portions of food. In addition, English *et al.*^(^^25^^)^ reported that the images of HED foods increased activation of areas thought to be involved in reward and taste processing compared with the images of LED foods. Against expectations, the authors of this study found an inverse correlation between the eating trait enjoyment of food and brain activation in response to the images of HED foods.

There is a complex relationship between portion size, energy density, liking and individual eating traits. Large portions seem to inhibit cognitive control, and HED items are especially attractive and rewarding compared with LED foods. Parents may, therefore, need to exert portion control for highly liked HED foods, especially for children with strong food approach tendencies. A complementary strategy to this is to encourage smaller size HED items and larger portions of nutrient-dense, low-energy items, which are well liked.

## Portion distortion

At a population level and of relevance to parents attempting to guide portion sizes, the landscape is challenging given recent trends for large portion sizes^(^^1^^–^^3^^)^. The US National Heart, Lung and Blood Institute has illustrated these trends for the US consumer by presenting the images of typical portions of commonly eaten foods from the 1950s and comparing these to the present day (https://www.nhlbi.nih.gov/health/educational/wecan/eat-right/portion-distortion.htm). Here the size and therefore energy content of a typical burger has increased from 1393·2 (333 kcal) to 2468·5 kJ (590 kcal), and for French fries from 878·6 (210 kcal) to 2552·2 kJ (610 kcal). The implication of this comparison is that consumers become familiar with the large portion sizes and judge this as normal or typical.

In the UK, the British Heart Foundation conducted interviews of 140 consumers using illustrations of meal items such as pizza and snacks such as chocolate and potato crisps. They found that consumers typically underestimated the number of servings in a package, for example, most (85 %) identified a medium-sized pizza as a single serving rather than as a serving for two and for a large bar of chocolate consisting of eight servings, most (73 %) estimated that it would provide four portions or fewer. It has been argued that exposure to large portions in the marketplace and elsewhere become normalised, consumers therefore experience an upward shift in portion size expectations^(^^26^^)^.

In a more systematic investigation than that conducted by consumer survey, Almiron-Roig *et al.*^(^^27^^)^ conducted a laboratory-based study with adults to examine portion size estimation of foods varying in unit number, meal type and energy density. In total, participants estimated the portion size of eleven foods at each of three visits, and they reported how this compared with their habitual intake. Portion size estimation was then compared with the reference value to produce an error of estimation. Across the thirty-three foods, a greater error of estimation was found for HED foods^(^^27^^)^, with the number of portions of HED foods overestimated. For beverages and LED items, the number of portions was underestimated. There was a greater error recorded for single-unit foods compared with multi-unit and snack foods. In all, the reference portion size was underestimated for a range of foods and beverages. This study illustrates lack of knowledge among adults for the reference or recommended portion size for commonly consumed foods and beverages, and has implications for how parents might judge portion sizes for their children.

Standard or reference portion sizes may be misjudged due to perceptual error. It may be harder to judge the amount or volume when faced with large portions^(^^28^^)^. Alternatively, judgement error may be attributable to habitual or personal norms exceeding social norms. Consumers may be unaware of reference portion sizes^(^^27^^)^ or the influence of large portions on food intake. Rolls *et al.*^(^^29^^)^ examined whether participants were aware of differences in portion size relative to habitual intake. In their study, sandwiches were provided in four sizes (6, 8, 10 and 12 inches) ranging in energy content from 2782·3 to 5585·6 kJ (665 to 1335 kcal). As expected, the larger sizes promoted greater intake supporting the PSE. When asked how the portions compared with their ‘typical’ portion size, participants rated all but the 6-inch sandwich as larger than their usual portion. Therefore, participants recognise that they had been served larger than normal portion sizes but this did not prompt adjustment in intake. It may be that the act of serving a large amount sets up an expectation of consuming a large amount. Keenan *et al.*^(^^30^^)^ conducted an experiment where they extracted planned portion sizes of three meal items from a computer-based task using the ‘method of constant stimuli’. They then offered participants one of these meal items (pasta in tomato sauce) in either a small or a large bowl; they then served themselves a portion into a second bowl (same size in both conditions). After eating until comfortably full, the image representing the ‘planned’ intake was presented and participants were asked whether they believed they had just eaten more or less than this amount, and by how much they had under- or overconsumed. If offered the large bowl to select from, participants ate more than their planned amount (demonstrating the PSE) and most correctly identified their overconsumption. However, when participants were asked to indicate by how much they had eaten above or below their initial plan, those who overconsumed after receiving a large portion underestimated their intake by 25 %. These results suggest that participants were aware that they had eaten more than planned but the magnitude of this error was underestimated. This suggests that planned intake can be disrupted by serving large portions and that the awareness of overeating is influenced by a judgement error.

The tendency to present large portions in the marketplace might influence PSE through an upward shift in what is considered normative. Providing large portions might promote a greater magnitude of error in judging portion size and consumers may misjudge the extent to which intake is influenced by how much is served.

What then can be done to encourage portion control and what downsizing strategies might be developed to counter the PSE? To address this question, the following discussion focuses on strategies for children since they are just beginning to learn about portion size and to establish eating habits.

## Downsizing for children: starting with snacks

Given that energy density and palatability are drivers of the PSE (Fig. 1), an obvious place to start for downsizing strategies is in the domain of snacking. Research with parents reveals that a number of portion control strategies are already applied to snacks for children^(^^31^^)^. These efforts are related to the concept that a snack is ‘something small’, and therefore needs to be portioned out accordingly^(^^32^^)^. Portioning snacks from a family bag involves using small containers, measuring cups and scales; or subdividing large adult or family portions, buying pre-packaged individual snacks, using hand measurements to gauge child size portions and letting children determine portion size^(^^32^^)^. Parents report lack of confidence in quantifying portions and that adjusting portions for children is both effortful and inconvenient^(^^33^^)^. Moreover, parents of pre-school children from low-income, urban households regard snacks as a means to curb appetite, not as sustenance, and as a means of behavioural control due to their high hedonic appeal^(^^31^^)^. Therefore, offering highly liked snack foods is complicated by emotional issues around providing treats to children and also by the confidence needed by parents to make dietary changes for their children^(^^33^^)^. It has been reported^(^^32^^)^ that many parents do not think about the portion size of snacks, but instead rely on situational cues such as the size of pre-packaged foods. Providing guidance to parents about portion size on packaging and labelling may benefit parents who rely on situational cues to determine the amount offered to children. Such labels should provide information on the appropriate amount to give to children at different ages and stages of development. In the UK, the EatWell guide (2016) advocates that foods high in sugar, fat and salt should be eaten less often and in small amounts. Practical guidelines have been issued by the Children's Food Trust ‘Eat Better, Start Better’ (2012) aimed at the early years (age 1–5 years) and at providers in day care centres and childminders in England. These voluntary guidelines suggest 40 g portions of fruit and vegetables and 50 g portions for cakes and desserts that contain fruit such as muffins or flapjacks. For foods such as potato chips and chocolate, the guide suggests that these foods should be avoided: ‘Sweet foods like cakes, biscuits, sweets and dried fruit should not be given as snacks as these can cause tooth decay. Instead, provide starchy foods and fruit or vegetables. Avoid salty snacks such as crisps’ (http://www.childrensfoodtrust.org.uk/). For those who are unable or unwilling to exclude these foods from the diets of children, it is not clear what would be considered an appropriate portion size.

A survey published by the Infant and Toddler Forum involving 1000 UK parents found that 79 % offer larger than recommended portions when serving meals, drinks and treats. For potato crisps, more than one-third of parents offered a whole bag (adult portion) to their toddler, and for chocolate buttons, 21 % of parents offered a whole bag which is 2·5 times the recommended serving. In a recent Scottish survey of maternal and child nutrition, 29 % of infants aged up to 12 months were offered at least one treat food daily such as chocolate, salty snacks and ice cream. Younger caregivers (aged 20–25 years) were more likely to offer these foods daily compared with older caregivers (aged over 35 years). More importantly, those infants from the most deprived backgrounds were more likely to be offered treat foods compared with those from the least deprived backgrounds. Although this report does not tell us about how much was served to the infant, it is likely that package sizes are used for convenience. The survey highlights that very early exposure to HED snacks occurs in some groups and the implication is that HED snacks given daily might be one of many contributing factors to the social gradient of early overweight and obesity (http://www.gov.scot/Resource/0053/00531610.pdf).

As a guide for parents, the UK-based Infant and Toddler Forum has developed a table of portion sizes including snack foods (https://www.infantandtoddlerforum.org/portion-sizes-table-2015). They advise that biscuits, cakes or puddings should be avoided in the under 2 years but included no more than once daily for the over 2 years; and for confectionery, sweet drinks and savoury snacks, these should be limited to occasional meal times, no more than once per week. In addition, the Infant and Toddler Forum provides a colour guide with notional portion sizes for children aged 2–4 years for a range of foods including both meal items and snacks. This resource provides a creative and engaging guide for parents. Whether the guide is effective in influencing behaviour is as yet unknown.

It is important to understand the implications of adjusting portion sizes served to children and a pilot study is currently underway to investigate the feasibility and acceptability of two downsizing strategies; reduction, whereby caregivers are instructed to reduce all HED snacks by 50 % and replacement which involves replacing all HED snack with fruit and vegetables (ClinicalTrials.gov NCT03339986). In the meantime, Public Health England has issued guidance to parents to suggest snacks be given in 418·4 kJ amounts and no more than two of these daily (https://www.gov.uk/government/news/phe-launches-change4life-campaign-around-childrens-snacking). Again, it is critical to evaluate the success of this campaign and its potential impact on behaviour change.

Given the appeal and HED of some snacks, and their status as treats in many households, it is unrealistic to expect these foods to be omitted from children's diets. Therefore, it is important to provide parents with portion size guidance and to investigate whether downsizing is an acceptable strategy, particularly as public health campaigns recommend smaller, medium-sized snacks for children.

## Downsizing for children: dealing with meals

It is even more complex to identify downsizing strategies for meal items, since children's energy requirements vary not only by age, but also by sex and activity levels. Nevertheless, efforts have been made to offer guidance to consumers (adults and children) in the proportions of food groups served within a meal.

In the USA, the 2010 dietary guidelines were translated into a visual My Plate design depicting the relative proportion of meal items on the plate (https://www.choosemyplate.gov/). This graphic representation shows that half of the plate should contain fruit and vegetables. There have been few studies to evaluate the effectiveness of simple messages about optimal meal proportions to influence intake^(^^34^^)^. In young adults, a novel plate design with portion size inserts produced a decrease in overall food intake but also reduced vegetable intake^(^^34^^)^. In contrast, Savage *et al.*^(^^35^^)^ varied the size of macaroni and cheese at lunch from 100 to 400 g and found that offering smaller, age-appropriate portions of the main energy-dense meal item significantly increased intake of LED items such as green beans and unsweetened applesauce. These authors argued that serving large amounts of highly palatable, energy-dense items suppresses intake of the less well-liked items. Therefore, strategies that downsize highly liked, energy-dense meal components low in nutrient density may promote dietary variety and quality of the meal consumed, while reducing the overall energy content. Variety could be used strategically as an adjunct to downsizing since it is known to promote intake, and like the PSE, is a reliable effect observed across age groups and eating environments^(^^36^^–^^38^^)^. Within the context of vegetable consumption, an increase in the provision^(^^39^^–^^41^^)^ and variety of vegetables may lead to increased vegetable intake in children^(^^42^^)^. As long as the variety offered is well liked, this technique could be used to offset smaller HED meal items, meaning that downsized HED items are balanced against a variety of palatable LED items to ensure children eat well and are satisfied.

Overall, these studies suggest that portion control aids and strategic use of variety may encourage children to achieve a higher proportion of intake from nutrient-rich, LED foods (Fig. 1). This proposal is plausible since there is evidence that adults can benefit from learning about portion control. In a study by Zuraikat *et al.*^(^^43^^)^, adults who had undergone extensive training in portion control still ate a greater amount of food from large portions, but they selected more of the LED items such as vegetables and reduced overall energy intake compared with those who had not received this training. These findings, in adults, reveal that portion control strategies may confer benefits to both overall energy intake and to the nutrient quality of the meal. Given that children are just beginning to establish eating habits, downsizing solutions may be acquired more quickly and easily than for adults, but this has yet to be tested.

## Changing social norms

As children develop, the influence of social factors such as their peer group becomes prominent and this has been demonstrated in laboratory settings^(^^44^^–^^46^^)^. In order to identify appropriate strategies for downsizing in adolescence, it is important to employ methods such as social media which are relevant and highly accessed by this age group. Social media has been promoted as a potential platform for behaviour change interventions^(^^47^^)^, and research has shown that a peer influence intervention may affect sexual health behaviour and knowledge^(^^48^^)^. Although social norms are known to be important in predicting dietary behaviour, little research has been carried out in adolescents. Studies are therefore needed to determine whether creative nudging methods can be applied to improving diet quality and reducing energy-dense snacks such as cakes, biscuits and sugary drinks. However, it is not yet known whether social media can be used to nudge behaviour towards selection of smaller portion sizes of HED items, including snacks, especially given both the palatability and popularity of these items among this age group.

## Packaging solutions for downsizing

As mentioned, parents rely on pre-packaged items and information on labels to guide portion control for children^(^^32^^)^. In order to make downsizing more convenient, improvements to food packaging can be used to influence consumer's purchase intention, buying behaviour and intake^(^^49^^)^. Consumers rely on package size, information and habitual use to determine serving sizes^(^^50^^)^. Attempts have been made to promote appropriate serving sizes through packaging design, such as smaller packaging and food labels with serving guidelines. However, recommended serving sizes are typically given for adults and packaging is not designed to reflect the age, stage or energy needs of different children. Therefore, it is not yet clear how parents might use packaging to assist with portion control. However, package design and information on recommended portion size to promote healthy eating, particularly for children, provides another potentially important downsizing strategy^(^^51^^)^.

## Discussion

The scientific evidence is robust that offering large portion sizes of energy-dense foods promotes energy intake^(^^5^^–^^13^^)^. A number of drivers have been proposed to account for the PSE^(^^5^^)^ (see Fig. 1). For example, consumers are guided by what is presented to them, using the amount served as a simple heuristic to determine intake, coupled with consumption norms and expectations developed over time and experience. However, the PSE is also influenced by relative palatability and is magnified by energy density (which is associated with palatability)^(^^12^^)^. Therefore, offering large portions of foods that are less liked mitigates against simple heuristics, in other words it is not only the size of the portion that matters but how much this food is liked. Given that the relative palatability of foods influences the PSE, simply offering large portions of LED items that are also nutrient dense may not in itself encourage intake to improve dietary quality and lower overall energy intake. Instead, the strategy of downsizing HED items and increasing portion size of LED items must take account of relative palatability. One implication of this proposal is that learning to like foods such as vegetables and fruit from childhood might encourage selection of these foods in greater proportions when offering downsized meal or snack items.

Consumers may misjudge appropriate sized portions for themselves and for their children, and this could be influenced by perceptual error. However, again the pivotal role of energy density is recognised since errors are greater for HED foods that are highly appealing^(^^27^^,^^28^^)^. Adults appear to be aware of the role of portion size on how much is eaten. They recognise large portions and realise that this increases intake but they underestimate the size of this difference^(^^30^^)^. Parents adjust portions of HED snacks in an effort to provide ‘something small’ for their children^(^^32^^)^, but frequency as well as amount must be considered in promoting a healthy diet. Even if children learn to accept small portions of HED items within a downsizing context, if these are eaten frequently, this will not result in a net benefit to their energy and nutrient intake.

Availability of food on the plate can be regarded as a conditioned stimulus, and indeed Kanoski and Davidson^(^^52^^)^ have suggested that overeating due to food cues can be considered within an associative learning analysis, in which energy regulation can be interpreted as a serial feature negative problem. According to this analysis, food cues predict appetitive post-ingestive reinforcement, but when satiety cues are also present, these should reduce the association between the food cues and post-ingestive reinforcement. That the PSE is curvilinear suggests this is a plausible account. However, in a single eating occasion, the lag between consumption and any emergent satiety cues means that consumers will continue to respond to available food cues, including the amount served. A way to counter this effect is to ensure that amounts served of HED foods are downsized to prevent overeating and are adjusted to the age and energy needs of the consumer.

An obvious and simple solution to the PSE is to offer small portions of HED snacks and meal items, particularly for children. Whether children and adolescents accept downsized portions without compensating elsewhere in the diet is not yet known. At least in the early years, there is the potential to set expectations of what size, volume and amount of food is appropriate within the context of a healthy diet, and to encourage a greater proportion of fruit and vegetable intake. Parents need clear guidance on portion sizes to promote appropriate consumption norms in the face of the robust nature of the PSE and the potential for perceptual errors in judging portion size, especially for HED snack and meal items. This guidance may take the form of visual aids on dishware, providing vegetables and fruit on half the plate, offering medium-sized, pre-packaged snacks and offsetting small portions with a variety of highly liked LED alternatives (see Fig. 1). Given the potential link between large portions, overconsumption and body weight status, practical recommendations to guide portion decisions are needed. However, creative solutions to downsizing place emphasis on individual families to control portions for children and adolescents. There is a considerable public support for the government working with businesses to develop products with fewer energy and in smaller portions as evidenced by Public Health England in their energy reduction initiative (https://www.gov.uk/government/publications/calorie-reduction-the-scope-and-ambition-for-action). If this initiative is combined with clearer labelling on portion/serving sizes for children and businesses produce smaller, pre-packaged ‘child size’ portions of foods, then together this will lead to changes in the obesogenic environment promoting downsized portions as the new consumption norm for HED foods and beverages.
